# Small extracellular vesicles have distinct CD81 and CD9 tetraspanin expression profiles in plasma from rheumatoid arthritis patients

**DOI:** 10.1007/s10238-023-01024-1

**Published:** 2023-02-24

**Authors:** Anne Rydland, Fatima Heinicke, Siri T. Flåm, Maria D. Mjaavatten, Benedicte A. Lie

**Affiliations:** 1 Department of Medical Genetics, University of Oslo and Oslo University Hospital, Oslo, Norway; 2 Institute of Clinical Medicine, University of Oslo, Oslo, Norway; 3 Division of Rheumatology, Diakonhjemmet Hospital, Oslo, Norway; 4 Department of Immunology, Oslo University Hospital, Oslo, Norway; 5 Center for Treatment of Rheumatic and Musculoskeletal Diseases (REMEDY), Diakonhjemmet Hospital, Oslo, Norway

**Keywords:** Extracellular vesicles, Rheumatoid arthritis, Methotrexate, Tetraspanins

## Abstract

Extracellular vesicles (EVs) are implicated in the pathogenesis of rheumatoid arthritis (RA) but little is known about the composition of specific small EV (sEV) subpopulations. This study aimed to characterize the CD63, CD81 and CD9 tetraspanin profile in the membrane of single EVs in plasma from treatment naïve RA patients and assess potential discrepancies between methotrexate (MTX) responder groups. EVs isolated from plasma were characterized using transmission electron microscopy, and detection of surface markers (CD63, CD81 and CD9) on single EVs was performed on the ExoView platform. All RA patients (*N* = 8) were newly diagnosed, treatment naïve, females, ACPA positive and former smokers. The controls (*N* = 5) were matched for age and gender. After three months of MTX treatment, responders (*N* = 4) were defined as those with ΔDAS28 > 1.2 and DAS28 ≤ 3.2 post-treatment. The isolated EVs were 50–200 nm in size. The RA patients had a higher proportion of both CD9 and CD81 single positive sEVs compared to healthy controls, while there was a decrease in CD81/CD9 double positive sEVs in patients. Stratification of RA patients into MTX responders and non-responders revealed a distinctly higher proportion of CD81 single positive sEVs in the responder group. The proportion of CD81/CD9 double positive sEVs (anti-CD9 captured) was lower in the non-responders, but increased upon 3 months of MTX treatment. Our exploratory study revealed distinct tetraspanin profiles in RA patients suggesting their implication in RA pathophysiology and MTX treatment response.

**Supplementary Information:**

The online version contains supplementary material available at 10.1007/s10238-023-01024-1.

## Background

The putative role of extracellular vesicles (EVs) in the pathogenesis, progression and treatment response of autoimmune diseases has gained increased focus [1–3]. EVs are membrane-derived nanoparticles that carry proteins, lipids, DNA and RNA, and are released by cells into biological fluids and tissues for intercellular communication. EVs can act as inflammatory mediators [4, 5] e.g., by being involved in the formation and distribution of immune complexes [6], T cell exhaustion [7] and cytokine-mediated signaling pathways [8].

Rheumatoid arthritis (RA) is an autoimmune disease that primarily manifests in synovial joints [9, 10], which can lead to irreversible damage if left untreated. The first line drug methotrexate (MTX) shows satisfactory response in 53–71% of patients [11]. Still, the large fraction of non-responding patients is likely related to underlying biological heterogeneity. Discovery of novel biomarkers might reduce trial-and-error time and enable personalized treatment of RA patients.

EVs represent a promising source of biomarkers in RA, as increased amounts of EVs have been reported in blood from RA patients [12–15]. Furthermore, subpopulations of EVs have been associated with both disease development and activity [14–18]. The majority of studies have used flow cytometry on platelet-poor plasma for bulk analysis of cluster of domain (CD) molecules on EVs (100–1000 nm). These studies have observed an increase in monocyte- (CD14 +), platelet- (CD41 + , CD61 +) [15, 17], endothelial cell- (CD146 +) [14], granulocyte- (CD66 +) [14], B cell- (CD19 +) [16] and T cell- (CD3 +) [18] derived EVs in RA compared to healthy controls. Changes in EV profiles after treatment with disease modifying anti-rheumatic drugs are evident, as a decrease in TNFα^+^ EVs was observed after four months of etanercept treatment [19], and a decrease in EVs from monocytes (CD14 +), platelets (CD41 +), endothelial cells (CD62 +), T cells (CD3 +) and B cells (CD19 +) was seen after four weeks on MTX, sulphasalazine and prednisone [16].

However, in addition to surface markers providing information of the cellular origin of EVs, they can also be characterized by their membrane bound proteins of the tetraspanin superfamily, including CD63, CD81 and CD9. The functionally important tetraspanins have a broad tissue distribution and are, surprisingly, found in higher concentrations on EVs compared to the cell of origin [20, 21]. Tetraspanins are involved in EV biogenesis, cargo selection, cell targeting, immune cell activation and cellular uptake of EVs [21, 22]. The EVs interact with each other or cellular transmembrane and cytosolic proteins through membrane microdomains enriched in tetraspanins [21]. Disease-specific alterations in tetraspanin profiles of EVs have been reported in cancer [23, 24] and infectious diseases [25, 26], but to our knowledge have not yet been investigated in RA or other autoimmune diseases.

**Fig. 1:**
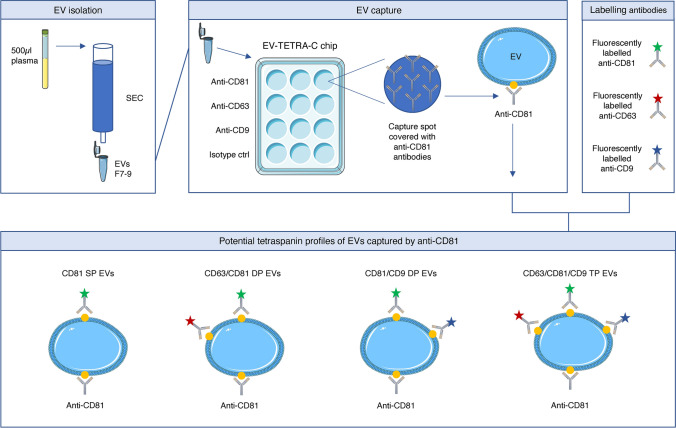
Workflow of the ExoView analysis from EV isolation to sEV profiling. EVs were isolated from plasma by size exclusion chromatography and subjected to ExoView analysis using the EV-TETRA-C chip. The lower panel shows potential tetraspanin profiles when capturing sEVs with anti-CD81, which is transferrable to sEVs captured with anti-CD63 and anti-CD9 although with some adjustments. The Figure was partly generated using Servier Medical Art, provided by Servier, licensed under a Creative Commons Attribution 3.0 unported license. *SP-single positive, DP-double positive, TP-triple positive

We hypothesized that certain EV subtypes, defined by their tetraspanin profile, might influence RA development and possibly MTX treatment response. To assess this, we performed an explorative study to investigate tetraspanin profiles of single EVs (Fig. 1) from RA patients and healthy controls. The RA patients were also investigated after ~ 3 months MTX treatment to assess potential alterations in EV tetraspanin profiles in response stratified patient groups.

## Methods

### Study participants

Eight patients diagnosed with RA according to the 2010 RA classification criteria [27] were recruited from Diakonhjemmet Hospital (*N* = 3), Lillehammer Hospital for Rheumatic Diseases (*N* = 3), Martina Hansen’s Hospital (*N* = 1) and Hospital of Southern Norway Trust (*N* = 1) through the Norwegian Very Early Arthritis Clinic (NOR-VEAC) observational study (ISRCTN05526276). At the time of inclusion RA patients were untreated and newly diagnosed, with MTX being prescribed. Two samples were collected from each patient; one prior to MTX treatment (pre-MTX) and one after approximately 3 months of MTX treatment (range: 3.25 ± 0.75, post-MTX). Upon inclusion, RA patients were clinically examined and parameters including anti-citrullinated peptide antibodies (ACPA) rheumatoid factor (RF), C-reactive protein (CRP), erythrocyte sedimentation rate (ESR), 28-point disease activity score (DAS28), smoking-status and body mass index (BMI) were recorded. RA patients from the NOR-VEAC cohort were divided into two groups based on their response to MTX treatment according to the EULAR response criteria [28]. Patients exhibiting a reduction in DAS28 score ΔDAS28 > 1.2 with a value of DAS28 ≤ 3.2 post-treatment were classified as responders (R), while patients with ΔDAS28 > 0.6 and ≤ 1.2 with post-treatment value ≤ 5.1 or ΔDAS28 ≤ 0.6 were classified as non-responders (NR).

The study also included age and gender matched healthy controls (*N* = 5, HC) recruited from the CFS/ME center at Oslo University Hospital. Information on BMI was available, however smoking status had not been recorded.

### Sample collection and processing

Peripheral blood was collected in Vacuette K_2_EDTA tubes and processed within 45 min. To remove cellular debris plasma samples were centrifuged at 1600–2200*g* for 10–15 min at room temperature, depending on the biobanking protocol of the recruiting hospital. Healthy control samples were additionally centrifuged at 15,000*g* for 15 min at 4 °C to eliminate large EVs and generate platelet-poor plasma. Samples were aliquoted and directly frozen, either at − 80 °C (*N* = 13) or at − 20 °C for one day then transferred to − 80 °C (*N *= 8).

### EV isolation

Blood plasma from the healthy controls was thawed at 4 °C and 500 µl platelet-poor plasma was transferred to a qEV original 70 nm column (Izon Science, Oxford, UK) for EV isolation by size exclusion chromatography (SEC). To ensure similar processing as the control samples, RA samples were thawed and centrifuged at 15,000*g* for 15 min at 4 °C prior to SEC. For all samples, the EVs were eluted in 500 µl filtered PBS per fraction and EV enriched fractions 7–9 were pooled, as according to the manufacturer’s recommendation. Freshly isolated EV aliquots were used for transmission electron microscopy (TEM), while EV aliquots used for ExoView analysis were stored at − 80 °C.

### Transmission electron microscopy

TEM was performed at the Department of Pathology core facility, Oslo University Hospital. SEC isolated EVs were subjected to TEM analysis by placing a 100 mesh hexagonal formwar carbon-coated copper grid (Electron Microscopy Sciences, Hatfield, PA) on 2.5–5 µl drops of EV suspension for 5 min. Incubation was followed by five washing steps on drops of distilled H_2_O before the grid was put on drops of 0.3% uranyl acetate in 2% methyl cellulose on ice for 5 min. Grids were removed from the uranyl acetate/methyl cellulose in stainless steel loops, and filter paper was used to absorb excess solution. Grids were air dried and examined using a Tecnai G^2^ Spirit TEM (FEI, Eindhoven, The Netherlands) equipped with a Morada digital camera using RADIUS imaging software.

### ExoView analysis

SEC-isolated EV samples were shipped on dry ice to NanoView, Malvern, UK where ExoView analysis was performed utilizing the EV-TETRA-C chip. This method combines single particle interferometric reflectance imaging sensing with antibody-based microchip capture and fluorescence detection to measure EV size and concentration, presence of EV tetraspanins and their colocalization profile. In short, the EV preparations were diluted according to the manufacturer's protocol and placed on microchips with separate wells coated with the capture probes; anti-CD63, anti-CD81 and anti-CD9 as well as anti-mouse IgG as isotype control. The microchips were incubated over night before three subsequent washing steps and incubation with a cocktail of fluorescent antibodies against CD63, CD81 and CD9, allowing for colocalization analysis of the three tetraspanins on single EVs (Fig. 1). This was followed by two additional washing steps. The ExoView R100 reader and nScan 2.8.19 acquisition software (NanoView) were used for imaging and data acquisition. Data analysis was performed using NanoViewer 2.8.10 (NanoView) with thresholds set to 50–200 nm. All measurements were done in triplicate.

### Statistics

Statistical analysis was performed using R version 4.2. All analyses included testing of normality by Shapiro–Wilk test prior to parametric or nonparametric analysis. Data following a normal distribution were submitted to ANOVA followed by Welch two sample *t*-test, to adjust for unequal variances, or Student’s *t*-test. Nonparametric tests included Kruskal–Wallis followed by Dunn’s test and Wilcoxon signed rank exact test. A *p*-value less than 0.05 was considered statistically significant.

## Results

### Characterization of EV populations in RA

The untreated, newly diagnosed RA patients included were all female and ACPA positive (Supplementary table 1). No significant differences (*p* > 0.1) were observed in age or BMI between RA patients and the gender matched healthy controls (Table 1).

**Table 1:** Summary of demographic and clinical characteristics of the study phenotypes

	Pre-MTX RA patients (*N* = 8)	Healthy controls (*N* = 5)
Female [in%]	8 [100]	5 [100]
Age at recruitment (median [range])	64 [50–72]	52 [48–59]
BMI (median [range])	27 [24–30]	23 [21–27]

**Fig. 2:**
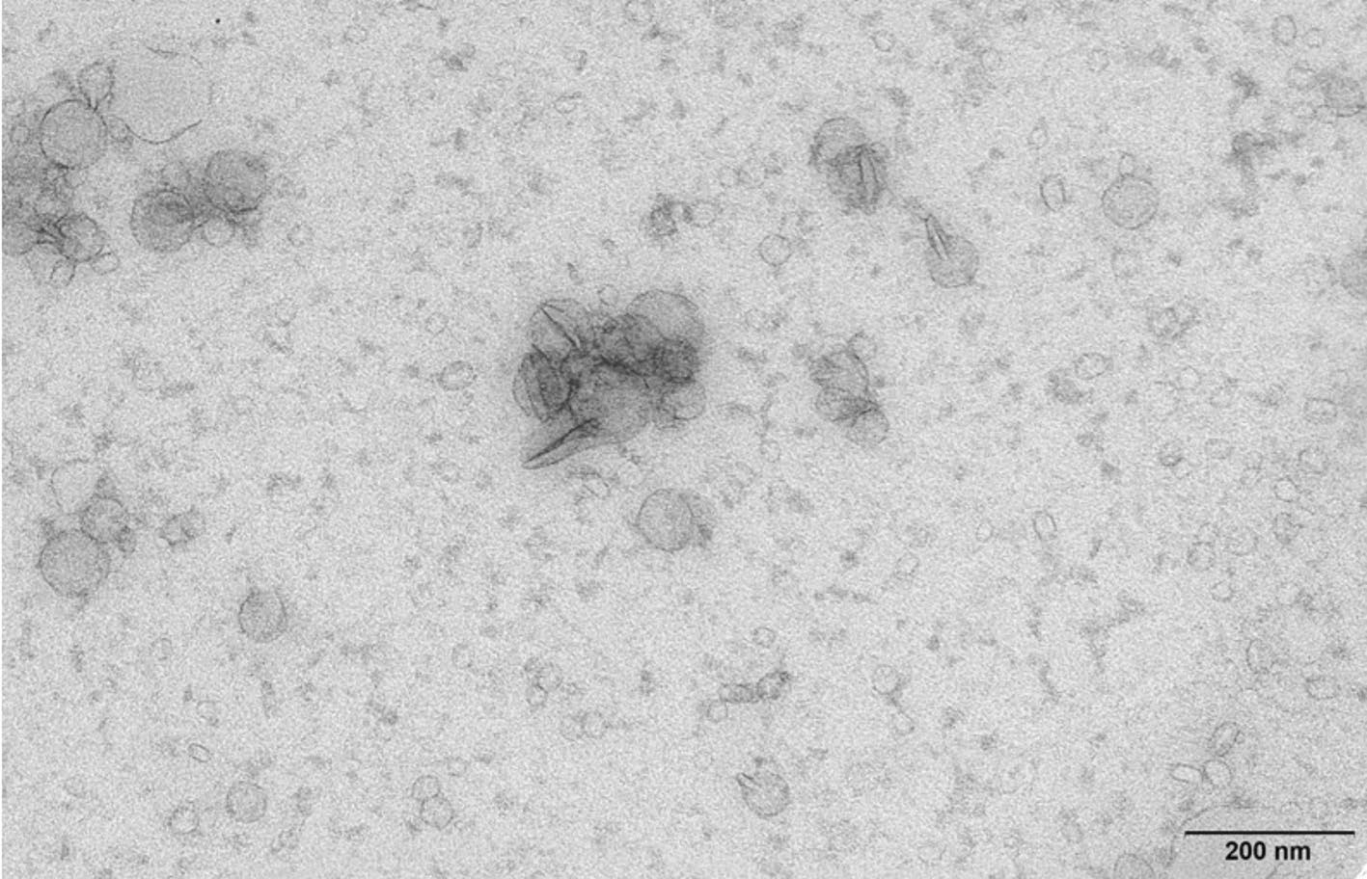
Characterization of SEC isolated plasma sEVs. Transmission electron microscopy micrograph of pooled sEV preparations

For both study phenotypes, the isolated plasma EVs ranged from 50 to 200 nm in diameter (outer limits of the ExoView analysis), with mean size 62 nm (± 17 nm) for RA patients and 57 nm (± 11 nm) for controls (Supplementary Fig. 1), characterizing them as small EVs (sEVs). This estimated size of the sEVs was confirmed by TEM analysis (Fig. 2).

**Fig. 3:**
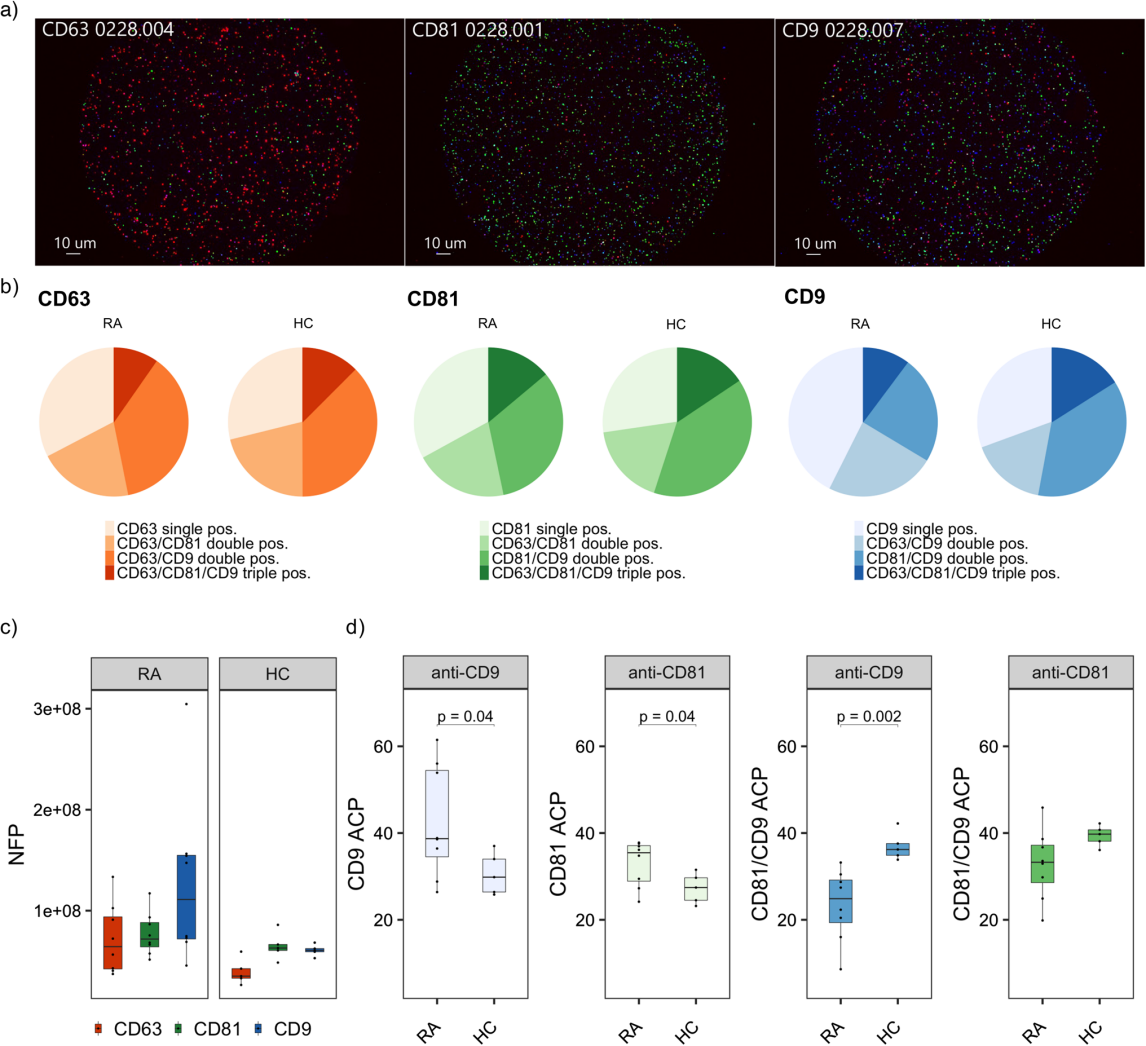
Single vesicle analysis of SEC isolated plasma sEVs. **a** Tetraspanin fluorescent staining of sEVs captured by anti-CD63 (red), anti-CD81 (green) and anti-CD9 (blue), **b** average ACP* distribution in RA patients and healthy controls for each tetraspanin capture probe, **c** number of fluorescent particles/ml captured by each capture probe, **d** phenotypic ACP analysis of CD9 single positive sEVs, CD81 single positive sEVs, anti-CD9 captured CD81/CD9 double positive sEVs and anti-CD81 captured CD81/CD9 double positive sEVs. For the analysis in **d** we compared the sample mean of the two study phenotypes using Welch’s two sample *t*-test for unequal variance. *ACP-Average colocalization percent, NFP-number of fluorescent particles/ml

We then assessed the presence of tetraspanins on the sEVs (Fig. 1) by capturing with either anti-CD63, anti-CD81 or anti-CD9 and measuring the number of fluorescent particles/ml (Fig. 3a, b). The profiles of RA patients differed from healthy controls, with the highest concentration of particles being captured by anti-CD9 in RA, in contrast to anti-CD81 in the healthy controls (Fig. 3c). Few sEVs appeared to carry CD63 in both phenotypes. Overall, the highest number of fluorescent particles captured by each antibody was observed in RA patients.

Next, we investigated the presence of two tetraspanins simultaneously on each single sEV by colocalization analysis which revealed distinct profiles between RA patients and controls for anti-CD81 and anti-CD9 captured sEVs (Fig. 3d), but not for anti-CD63. Significantly more sEVs only expressed CD9 (*p* = 0.04) or CD81 (*p* = 0.04) on their surface in RA patients compared to healthy controls. In contrast, CD81/CD9 double positive sEVs, captured by anti-CD9, was significantly reduced in RA patients (*p* = 0.002). A similar trend was observed for anti-CD81 captured sEVs, but this difference did not reach statistical significance (*p* = 0.06).

Further analysis of sEVs carrying all the tested tetraspanins revealed a reduced amount of triple positive sEVs in RA only for anti-CD9 captured sEVs (*p* = 0.03) (Supplementary Fig. 2). This adds to the observed lack of involvement of CD63 in RA as this difference probably is related to the previous findings of anti-CD9 captured sEVs.

### MTX treatment responders had distinct sEV profiles

Patient samples collected after approximately 3 months (3.25 ± 0.75 months) of MTX treatment were included in the analysis to investigate changes in the sEV population. Clinically, half of the patients were responders (ΔDAS28 > 1.2 and DAS28 ≤ 3.2 post-treatment) while the remaining patients were clear non-responders (Table 2, Supplementary table 1). We compared these two distinct responder groups to account for the limited sample size.

**Table 2:** Clinical features of MTX responders and MTX non-responders

	MTX responders (*N* = 4)	MTX non-responders (*N* = 4)
DAS28 at baseline (median [range])	5.1 [4.3–6.7]	4.4 [4.2–5.1]
DAS28 post MTX treatment (median [range])	2.7 [2.1–3.1]	4.5 [4.1–5.2]
ΔDAS28 (median [range])	2.5 [1.5–4.2]	-0.01 [-0.2–0.1]
ACPA positive [in %]	4 [100]	4 [100]
RF positive [in %]	3 [75]	3 [75]
Former smokers [in %]	4 [100]	4 [100]

Listed in the table are the 28-point disease activity score (DAS28) at baseline and after MTX treatment 3.25 ± 0.75 months, change in DAS28 (ΔDAS28), anti-citrullinated peptide antibodies (ACPA), rheumatoid factor (RF) and smoking-status.

**Fig. 4:**
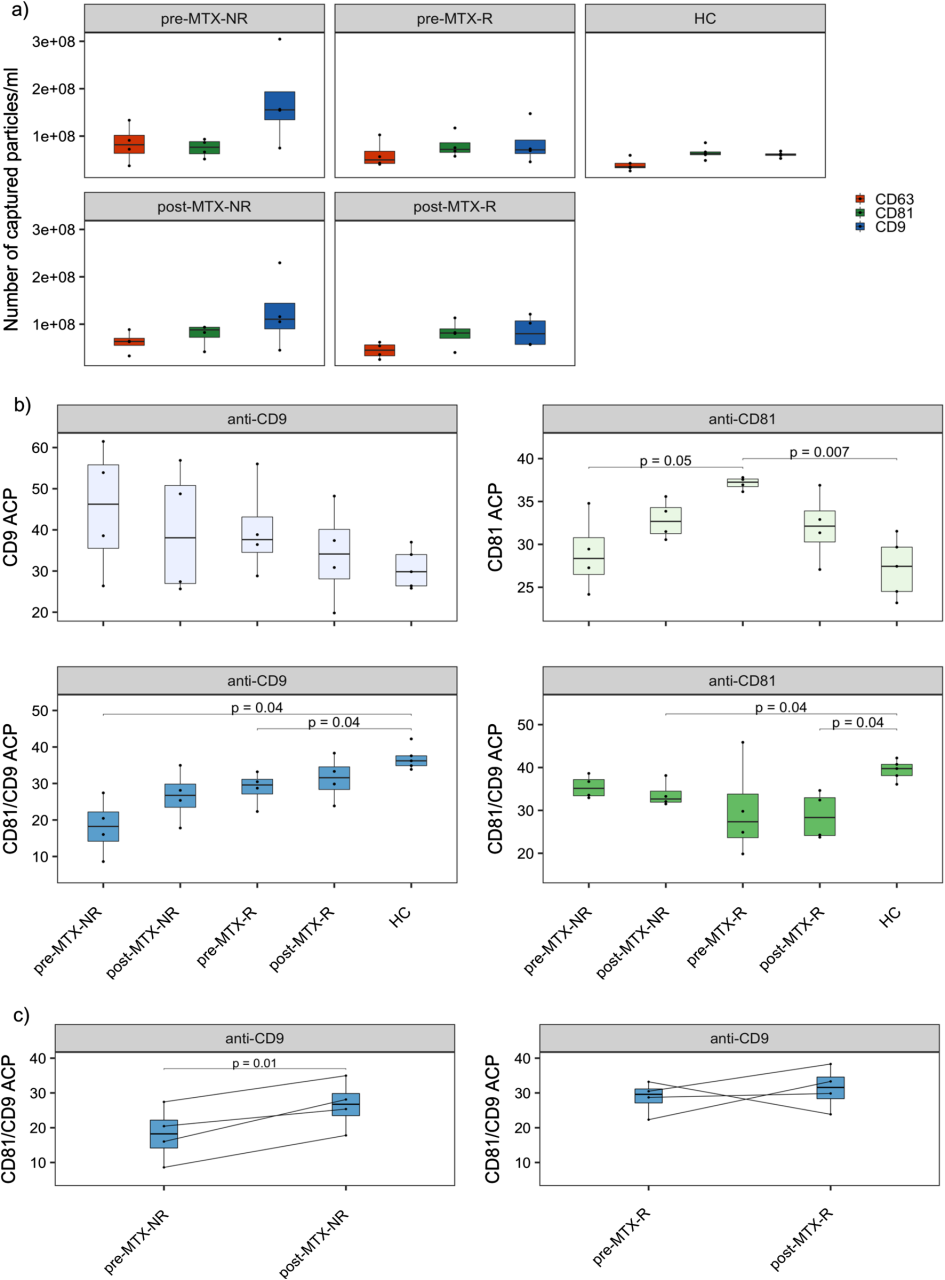
Stratified colocalization and capture analysis of RA patients and healthy controls. **a** number of fluorescent particles/ml captured by each capture probe in the five groups, **b **relative distribution of CD9 single positive sEVs, CD81 single positive sEVs, CD81/CD9 double positive sEVs captured by either anti-CD9 or anti-CD81, **c** paired analysis of CD81/CD9 double positive (anti-CD9 captured) sEVs in non-responders and responders pre- and post-MTX. We compared the sample mean of the groups using Welch’s two sample *t*-test for unequal variance. *ACP- average colocalization percent, NFP-number of fluorescent particles/ml

Stratification according to treatment response revealed that the difference detected in RA patients prior to treatment (Fig. 3c) could largely be attributed to the non-responders, as they displayed the highest concentration of CD9 captured sEVs (Fig. 4a). This overrepresentation of sEVs captured by CD9 was maintained after treatment. The responders, on the other hand, showed similar amounts of CD9 and CD81 captured sEVs, as did the controls.

Assessment of sEVs only expressing one of the three tetraspanins revealed that responders had a significantly higher proportion of CD81 single positive sEVs than non-responders (*p* = 0.05) and healthy controls (*p* = 0.007) (Fig. 4b). The level of CD81 single positive sEVs was reduced after treatment in the responders, while an opposite trend was seen in the non-responders.

When investigating sEVs positive for both CD81 and CD9, the highest proportion was seen in healthy controls and the lowest in non-responders pre-MTX (*p* = 0.04) when captured with anti-CD9 (Fig. 4b). This co-expression was also reduced in the MTX responders before treatment compared to healthy controls (*p* = 0.04). Although the relative expression of these CD81/CD9 double positive sEVs was low in both groups post-treatment, they did not differ significantly from healthy controls. RA patients also had a lower relative expression of CD81/CD9 double positive anti-CD81 captured sEVs than healthy controls, but for this sEV population the differences were only significant between the post-MTX groups and healthy controls (*p* = 0.04) (Fig. 4b).

We also assessed the change in CD81/CD9 double positive sEVs captured by anti-CD9 in the RA patients before and after MTX treatment (Fig. 4c). In both responders and non-responders, we saw an increased number of CD81/CD9 double positive sEVs after MTX treatment for the majority of patients, but this was only significant among non-responders (*p* = 0.01) and not the responders (*p* = 0.6).

## Discussion

In this study, we observed distinct distributions of CD9 and CD81 tetraspanins on sEVs, as the RA patients had more sEVs carrying only one of these markers, while healthy controls to a larger extent had sEVs with both these membrane proteins. Furthermore, we observed a discrepancy between MTX responders and non-responders, with responders having a unique high relative proportion of CD81 single positive sEVs.

To date, the well-established EV specific tetraspanins CD9, CD63 and CD81 have so far only been used in RA studies as qualitative control markers to demonstrate the bulk presence of EVs [29]. Hence, to our knowledge, this is the first study characterizing the tetraspanin profile of single EVs in RA patients including possible changes in sEV profile associated with MTX treatment response. The main limitations of our study were the sample size influencing our statistical power and the lack of smoking status for the controls. However, our results motivate further single EV studies in RA, which is interesting given the leap in knowledge of pathogenic cell types provided by recent single cell studies.

The low relative proportion of CD81/CD9 double positive sEVs (anti-CD9 captured) observed in RA patients, which was lowest in non-responders at baseline and significantly increased after MTX treatment, indicates that MTX might influence the expression of this sEV subpopulation. Although MTX suppresses inflammation, the exact functions are largely unknown. A study on sEVs from a synovial cancer cell line revealed that MTX modified the sEV proteome by increasing levels of immunosuppressive and anti-oxidant proteins, and that MTX suppressed several of the IL-1β-induced pro-inflammatory changes of the sEV proteome [30]. Together with our results, this suggests that MTX treatment affects the composition of sEV populations.

Interestingly, RA patients who responded well to MTX had a distinct, high prevalence of CD81 single positive sEVs at baseline compared to non-responders and controls. This indicates a potential role of CD81 single positive sEVs in the efficacy of MTX in RA patients. The ExoCarta database [31] recognizes close protein–protein interaction of CD81 with several other proteins identified in EVs including TSPAN4, KIT, ITGA4, CR2, IFITM1 and CD19 which are all also found in immune cells according to the Human Protein Atlas [32]. More is known about the proteins’ functions on a cellular level than on EV level. Still, their function on cells might, to some extent, be transferrable to EVs. In B cells, CD81 directly interacts with CD19 and, together with CD21, they make up the B cell co-receptor complex. Upon B cell activation, CD81 appear to dissociate from CD19 in order to allow CD19 to interact with the B cell receptor [33]. B cells are known to be involved in RA pathogenesis and changes in B cell receptor activity, through the release of CD81 on EVs, may be implemented in RA.

In cells, CD81 and CD9 associate with A Disintegrin and Metalloprotease domain-containing protein 10 (ADAM10), among other proteins, by either forming separate complexes or by incorporation of ADAM10 into tetraspanin-enriched microdomains [34]. ADAM10 is involved in regulating antibody production and inflammatory responses. Cleavage of its substrates, e.g., TNF-α, CXCL16 and EGF, in synovial tissue leads to increased pro-inflammatory activity, which coincides with the increased expression of ADAM10 observed in synovial tissue of RA patients [35, 36]. ADAM10 is also present in EVs, and further studies are needed to reveal whether interactions of these proteins also play a role at the EV level for RA inflammation and treatment response.

Our explorative study revealed RA and MTX treatment response specific tetraspanin profiles of plasma derived sEVs. Even though our sample size was limited, yet phenotypically homogenous, our novel and significant findings after assessing membrane proteins on single EVs were in line with biological data. Our findings warrant validation in larger cohorts, and future insights into the biological roles of tetraspanins on the EV membrane may elucidate their functions in RA.

### Supplementary Information

Below is the link to the electronic supplementary material.

Supplementary file1 (PDF 1416 kb)

## Data Availability

The datasets used during the current study are available from the corresponding author on reasonable request.

## Abbreviations

EVs: Extracellular vesicles
RA: Rheumatoid arthritis
MTX: Methotrexate
ACPA: Anti-citrullinated peptide antibodies
RF: Rheumatoid factor
CRP: C-reactive protein
ESR: Erythrocyte sedimentation rate
DAS28: 28-Point disease activity score
HC: Healthy control
SEC: Size exclusion chromatography
TEM: Transmission electron microscopy
sEVs: Small extracellular vesicles
