# Supplementary material for: Small extracellular vesicles have distinct CD81 and CD9 tetraspanin expression profiles in plasma from rheumatoid arthritis patients
Source: Clin Exp Med. 2023 Feb 24;23(6):2867–75. doi: 10.1007/s10238-023-01024-1 (PMC10543154; doi:10.1007/s10238-023-01024-1)
Supplement: Supplementary file 1 — Supplementary file1 (PDF 1416 kb) [file 10238_2023_1024_MOESM1_ESM.pdf]

SUPPLEMENTARY MATERIAL

Supplementary table 1. Characteristics of RA patients. All RA patients were female, $ACPA^+$ and former smokers.

RA patient	*Age*	*BMI*	*Months on MTX*	*DAS28 at* baseline	*DAS28 at follow up*	*∆DAS28*	*CRP at baseline*	*CRP at follow up*	*ESR at baseline*	*ESR at follow up*	*Response to MTX*
1	72	24.8	2.5	6.7	2.6	4.1	112	12	74	22	R
2	60	29.4	3.3	4.7	3.2	1.5	17	4	41	26	R
3	50	28.2	3.9	4.3	2.1	2.2	2	1	42	14	R
4	62	25.9	3.0	5.6	2.9	2.7	7	8	37	23	R
5	68	23.8	2.7	4.3	4.5	-0.2	3	18	29	56	NR
6	65	27.4	3.2	5.1	5.2	-0.1	23	19	47	42	NR
7	66	25.2	3.4	4.5	4.5	0	4	9	20	21	NR
8	50	29.8	4	4.2	4.1	0.1	19	21	17	22	NR

a)

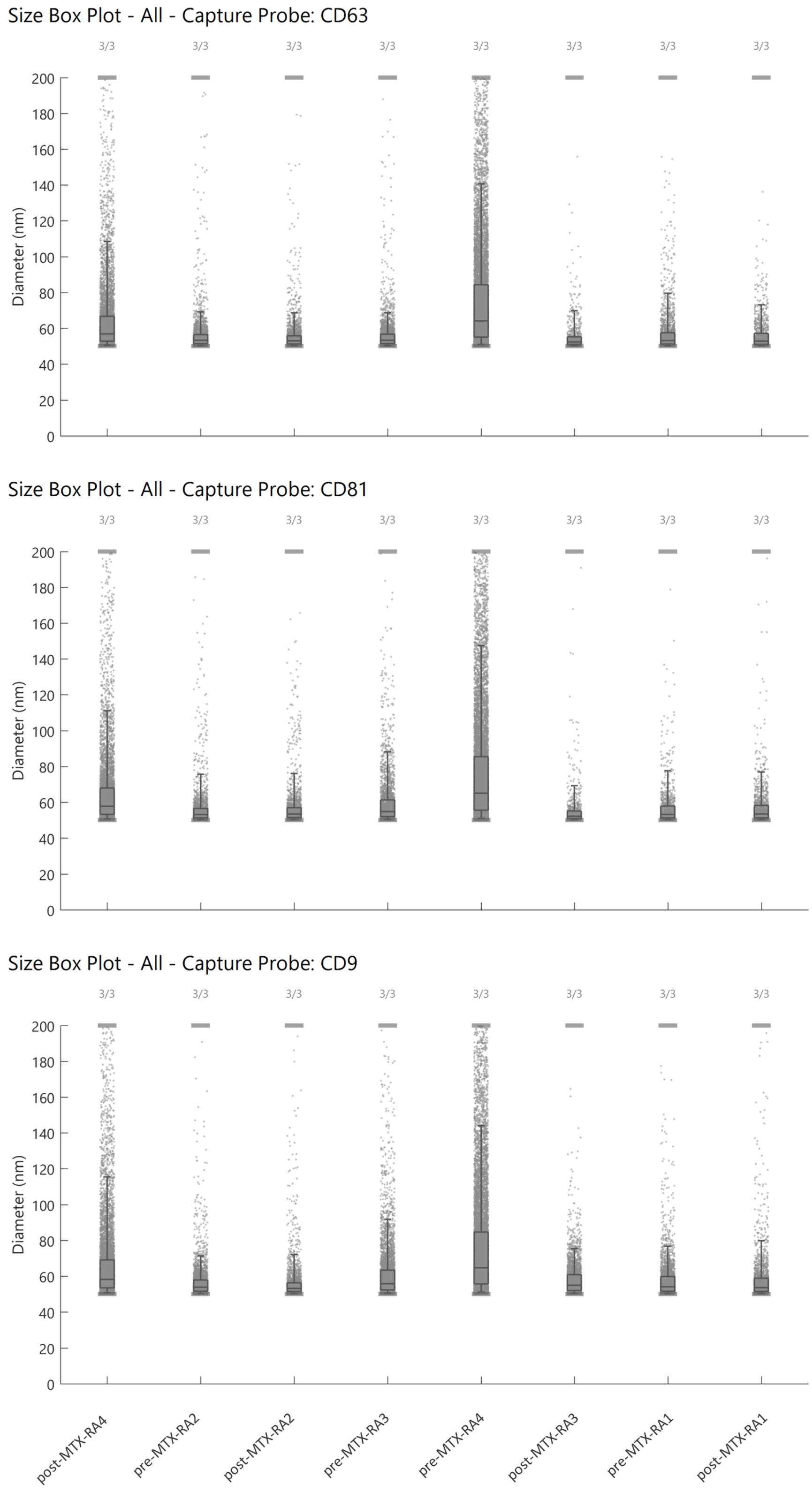
Size Box Plot - All - Capture Probe: CD63
Size Box Plot - All - Capture Probe: CD81
Size Box Plot - All - Capture Probe: CD9
3/3
Diameter (nm)
0
20
40
60
80
100
120
140
160
180
200
post-MTX-RA4
pre-MTX-RA2
post-MTX-RA2
pre-MTX-RA3
pre-MTX-RA4
post-MTX-RA3
pre-MTX-RA1
post-MTX-RA1

b)

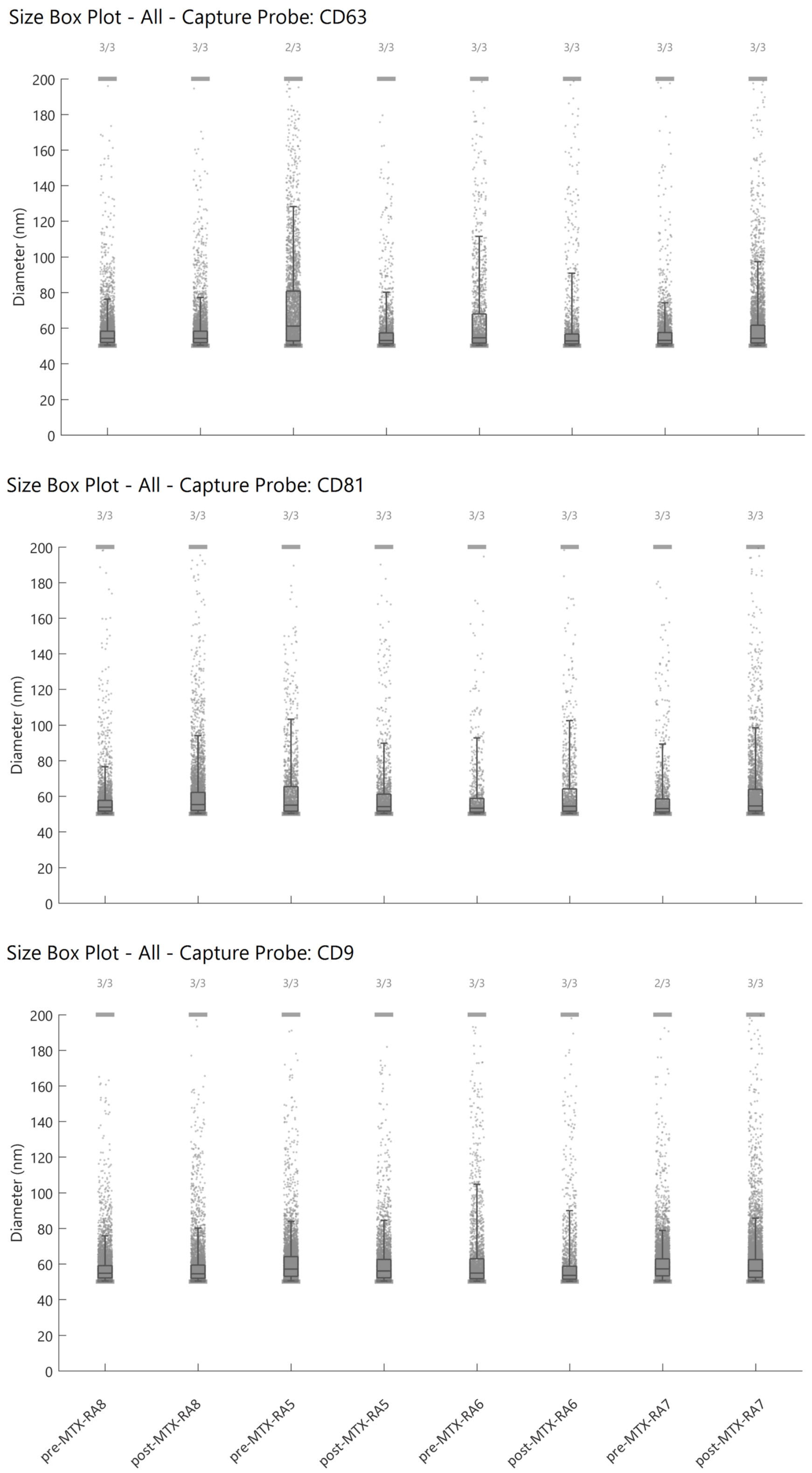
Size Box Plot - All - Capture Probe: CD63
3/3
3/3
2/3
3/3
3/3
3/3
3/3
3/3
Diameter (nm)
0
20
40
60
80
100
120
140
160
180
200
Size Box Plot - All - Capture Probe: CD81
3/3
3/3
3/3
3/3
3/3
3/3
3/3
3/3
Diameter (nm)
Size Box Plot - All - Capture Probe: CD9
3/3
3/3
3/3
3/3
3/3
3/3
2/3
3/3
Diameter (nm)
pre-MTX-RA8
post-MTX-RA8
pre-MTX-RA5
post-MTX-RA5
pre-MTX-RA6
post-MTX-RA6
pre-MTX-RA7
post-MTX-RA7

c)

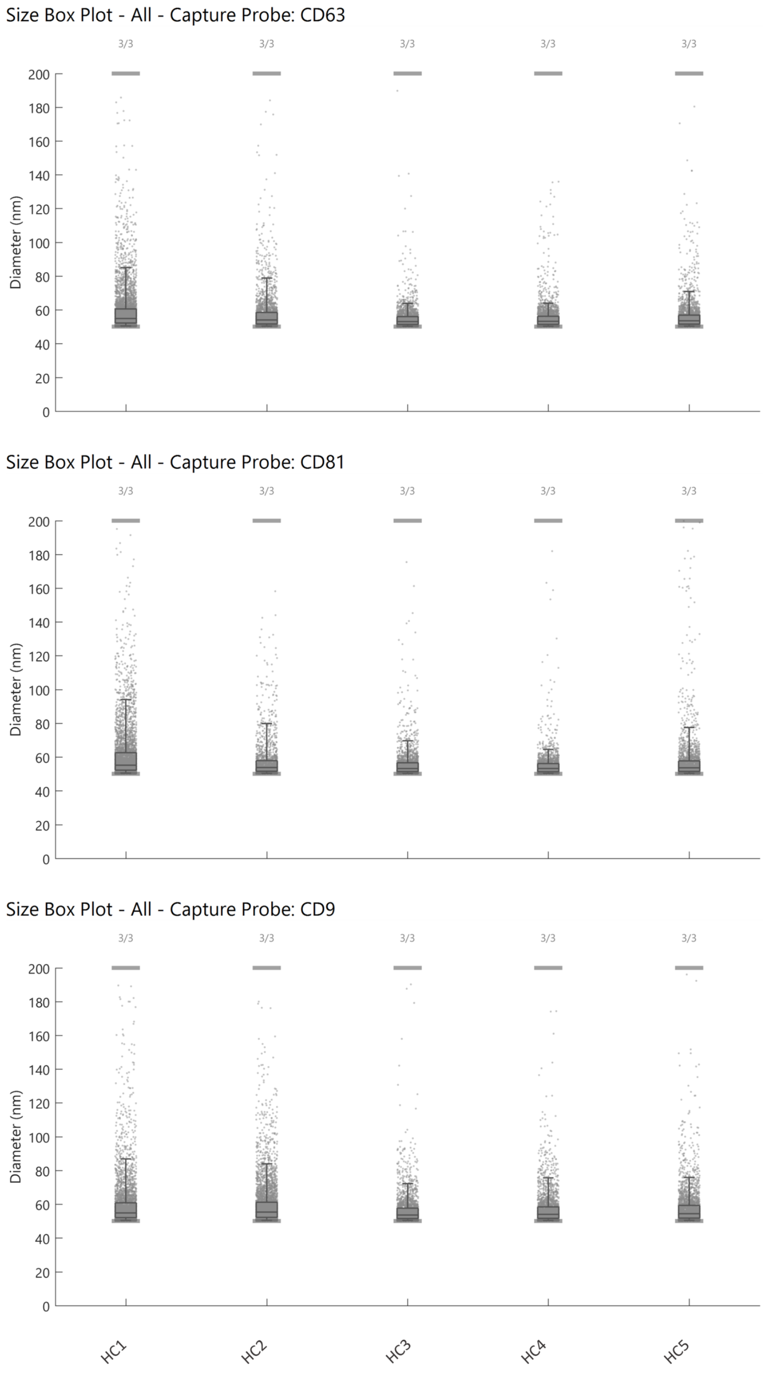

Supplementary figure 1. Size distribution of sEVs captured by the ExoView chip. a) MTX-responders, b) MTX-non-responders and c) healthy controls.

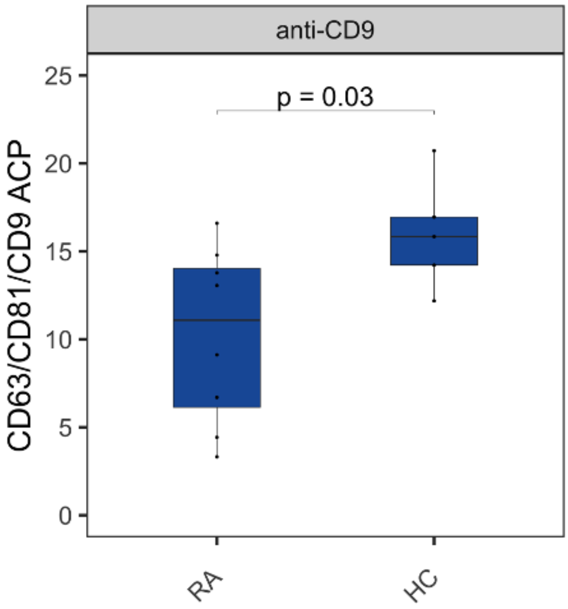

Supplementary figure 2. Phenotypic analysis of triple positive sEVs captured by anti-CD9. The average colocalization percent (ACP) sample mean of the two study phenotypes were compared using Welch's two samples t-test.
